# ICU physicians’ and internists’ survival predictions for patients evaluated for admission to the intensive care unit

**DOI:** 10.1186/s13613-018-0456-9

**Published:** 2018-11-14

**Authors:** Monica Escher, Bara Ricou, Mathieu Nendaz, Fabienne Scherer, Stéphane Cullati, Patricia Hudelson, Thomas Perneger

**Affiliations:** 1 Pain and Palliative Care Consultation, Division of Clinical Pharmacology and Toxicology, Geneva University Hospitals, Rue Gabrielle-Perret-Gentil 4, 1211 Geneva, Switzerland; 2 Unit of Development and Research in Medical Education, Faculty of Medicine, University of Geneva, Geneva, Switzerland; 3 Division of Intensive Care, Geneva University Hospitals, Geneva, Switzerland; 4 Division of General Internal Medicine, Geneva University Hospitals, Geneva, Switzerland; 5 Division of Primary Care Medicine, Geneva University Hospitals, Geneva, Switzerland; 6 Division of Clinical Epidemiology, Geneva University Hospitals, Geneva, Switzerland

**Keywords:** Intensive care, Survival, Prediction, Patient admission, Triage

## Abstract

**Background:**

A higher chance of survival is a key justification for admission to the intensive care unit (ICU). This implies that physicians should be able to accurately estimate a patient’s prognosis, whether cared for on the ward or in the ICU. We aimed to determine whether physicians’ survival predictions correlate with the admission decisions and with patients’ observed survival. Consecutive ICU consultations for internal medicine patients were included. The ICU physician and the internist were asked to predict patient survival with intensive care and with care on the ward using 5 categories of probabilities (< 10%, 10–40%, 41–60%, 61–90%, > 90%). Patient mortality at 28 days was recorded.

**Results:**

Thirty ICU physicians and 97 internists assessed 201 patients for intensive care. Among the patients, 140 (69.7%) were admitted to the ICU. Fifty-eight (28.9%) died within 28 days. Admission to intensive care was associated with predicted survival gain in the ICU, particularly for survival estimates made by ICU physicians. Observed survival was associated with predicted survival, for both groups of physicians. The discrimination of the predictions for survival with intensive care, measured by the area under the ROC curve, was 0.63 for ICU physicians and 0.76 for internists; for survival on the ward the areas under the ROC curves were 0.69 and 0.74, respectively.

**Conclusions:**

Physicians are able to predict survival probabilities when they assess patients for intensive care, albeit imperfectly. Internists are more accurate than ICU physicians. However, ICU physicians’ estimates more strongly influence the admission decision. Closer collaboration between ICU physicians and internists is needed.

**Electronic supplementary material:**

The online version of this article (10.1186/s13613-018-0456-9) contains supplementary material, which is available to authorized users.

## Background

The decision to admit or not a patient to the intensive care unit (ICU) is often complex and is based on a combination of criteria [1]. They include a patient’s need for life-sustaining therapies, but also patient prognosis, prehospital functional status, patient preferences, and available clinical expertise and resources. Since expected survival benefit is one of the main justifications for ICU admission, physicians must estimate and compare the patient’s prognosis according to two scenarios: continued care on the ward, and care in the ICU. Most scoring systems apply to patients once admitted to the ICU and the low performance of the few existing triage scores limits their use [2]. Moreover, even validated scoring systems are not accurate enough in predicting an individual’s mortality and they should not be used alone to determine level of care [1, 3, 4].

Previous research has shown that physicians predict patient survival more accurately than scoring systems [5]. However, their accuracy in prediction was generally assessed for patients already admitted to the ICU. In one French study, ICU physicians estimated patient mortality at the time of the admission decision [6]. Their predictions were higher than the observed mortality rates both for non-admitted and for admitted patients. Furthermore, the requesting physicians and the ICU physicians sometimes disagree about the appropriateness of an ICU admission [7, 8]. Whether this is due to differences in survival estimates or to other reasons is currently unknown.

When medical in-patients become critically ill, the need for intensive care is first assessed by the internists. They will then request an ICU consultation. The final decision about the patient’s admission to the ICU belongs to the ICU physician. The ICU physician is not involved in the patient’s care if he is not admitted to the ICU. The objective of this study was to compare internists’ and ICU physicians’ predictions of patient survival if cared on the ward or in the ICU, i.e., expected survival benefit from intensive care, and to determine whether the survival estimates correlated with the admission decisions and with observed survival. We hypothesized that agreement between the physicians would be weak to moderate, that expected survival would be greater in admitted than in non-admitted patients, and that observed survival would be associated with predicted survival. We also hypothesized that ICU physicians, who are used to care for acutely ill patients, would be more accurate in their predictions than internists.

## Methods

### Setting

The study took place at the Geneva University Hospitals, a tertiary care hospital of 1741 beds, including 156 internal medicine beds and 34 adult ICU beds, between August 2014 and August 2015. It was approved by the Geneva Research Ethics Committee.

### Participants

All consecutive ICU consultations for patients hospitalized in the Division of General Internal Medicine were identified. The internist and the ICU physician directly involved were eligible. Upon first contact, all physicians provided written consent to participate and socio-demographic data.

### Data collection

Internists and ICU physicians were contacted by phone within 12 h of the request for ICU consultation and were asked to complete a questionnaire. Questionnaires were administered orally, by phone, or by email, according to physicians’ preferences. Reminders were sent 3 days later when necessary. Physicians were asked to predict patient survival (i.e., “probability that the patient survives the acute health problem”) if admitted in the ICU and if staying on the ward using 5 predetermined categories of probabilities (< 10%, 10–40%, 41–60%, 61–90%, > 90%). They also rated how confident they were in their estimates on a Likert scale ranging from 1 (not at all confident) to 5 (fully confident). Internists were asked about the reason for requesting the ICU consultation and whether the patient was admitted or not. The ICU consultation was included and patient data were recorded only if both physicians completed the questionnaire. Patient data (gender, age, comorbidities) and mortality at 28 days were collected from the electronic patient file.

### Sample size and statistical analysis

Sample size was driven by the main objective of the study, i.e., modeling of the decision to admit a patient to intensive care. Assuming 8 potential predictors of the decision to admit the patient, we intended to enroll 80 patients in the smaller of the 2 groups (admitted or not), and aimed for 160 patients if the admission rate was 50%. As the observed admission rate was 70%, the sample size was increased to 200.

Each patient was assigned 4 survival estimates (2 by each of the physicians: survival on the ward and in the ICU) on a 5-point ordinal scale. We compared survival predictions on the ward versus in the ICU for each physician, and survival predictions by the internist versus the intensivist for each location, using paired Wilcoxon tests. We also obtained Spearman correlation coefficients between survival predictions.

To capture the expected survival benefit of an ICU admission, we computed the difference, in survival categories, between predicted survival in the ICU and on the ward, for each physician (thus 0 indicated the same survival category in both locations, and 4 a survival > 90% in the ICU and < 10% on the ward). We cross-tabulated this expected survival benefit with the admission to the ICU, expecting that patients with the highest expected benefit would be the most likely to be admitted (Table 3).

To clarify which physician’s opinion weighed more in the decision to admit the patient, we used a logistic regression model, with admission as the dependent variable, and expected survival benefit estimated by each physician as covariates (because of sparse data, expected survival benefit was grouped as − 1 or 0, 1 or 2, and 3 or 4).

To assess the accuracy of physicians’ predictions, we cross-tabulated predicted survival with observed survival at 28 days, stratifying on the admission decision, separately for the 2 categories of physicians. Because expected survival was on an ordinal scale, we obtained *P* values for linear trend (Table 4). We also obtained areas under the receiver operating characteristic (ROC) curves. The area represents the probability that predicted survival would be lower for a randomly selected patient who died than for a randomly selected patient who survived; 0.5 corresponds to a coin toss, and 1.0 to perfect discrimination. To clarify which physician’s survival prediction was more accurate, we used a logistic regression model with actual survival as the dependent variable, and the 2 physicians’ predictions as independent variables.

Finally, we computed mean ratings of confidence (on a 1–5 scale) in the four survival estimates and compared them across levels of survival using Kruskal–Wallis tests.

## Results

During the study period, 219 patients were assessed for intensive care admission and 201 situations were included. They involved 128 men and 73 women (Table 1). Mean age was 64.9 years (SD 14.3). Among the patients, 140 (69.7%) were admitted to the ICU and 58 (28.9%) died within 28 days.

**Table 1:** Patient characteristics

Characteristics	Patients, N (%)^a^ (*n* = 201)
Men	128 (63.7)
Age, median (IQR) (year)	67 (56–77)
Living place	
Home	191 (95)
Nursing home	10 (5)
Advanced disease	105 (52.2)
Type of disease in patients with advanced disease^b^	(*n* = 105)
Metastatic cancer or active hematologic malignancy	37 (35.2)
Chronic obstructive pulmonary disease (FEV ≤ 50% or non-invasive ventilation or oxygenotherapy)	38 (36.2)
Chronic heart failure (NYHA III and IV and/or LVEF ≤ 20%)	7 (6.6)
Chronic renal failure (GFR ≤ 30 ml/min)	20 (19.0)
Cirrhosis Child B or C	18 (17.1)
Number of hospitalizations in previous 12 months	
0	107 (53.2)
1	39 (19.4)
> 1	55 (27.4)
Number of days between admission to general internal medicine wards and ICU consultation, median (IQR)	3 (1–8)
Code status (2 missing): full code	104 (51.7)
Reason for calling ICU^c^	
Respiratory failure	111 (55.2)
Cardiac failure or shock (including sepsis)	55 (27.4)
Neurological symptoms	32 (15.9)
Cardiac arrest or arrhythmia	16 (8)
ICU physician’s advice	48 (23.9)
Other	18 (8.9)

^a^Data are *N* (%) of patients unless otherwise indicated

^b^Total > 100% because more than one advanced disease per patient

^c^Total > 100% because more than one reason possible

In total, 30 ICU physicians (1–14 assessments per physician) and 97 internists (1–11 assessments per physician) participated in the study (Additional file 1: Table S1). ICU physicians were mostly men (66%) and internists mostly women (61%). ICU physicians were older than internists (mean age 38 vs. 30 years, respectively). They were more experienced: mean years from graduation were 12 and 7, respectively, and mean years of experience in medical specialty were 7 and 3.5, respectively. The delay between the admission decision and physicians’ prediction of survival was one day or less for 143 (71.1%) of the internists and 127 (63.2%) of the ICU physicians (McNemar test, *P* = 0.11).

### Predicting survival

The physicians used all five categories of survival probabilities (Table 2). Both physician groups rated expected patient survival significantly higher with care in the ICU rather than on the ward, e.g., the ICU physicians assigned 59.2% of patients to the 2 highest categories of survival (> 60%) if in the ICU, but only 24.8% to the same categories of survival on the ward. For the internists, the corresponding proportions were 67.9% and 13.9%. The internists were more optimistic about the patients’ chances of survival with intensive care than the ICU physicians (*P* = 0.006) and somewhat more pessimistic about survival on the ward, but the latter difference was not statistically significant (*P* = 0.079). As expected, the survival estimates were correlated with each other, both within physician (Spearman r correlation coefficient 0.69 for the ICU physician and 0.61 for the internist) and between physicians (0.48 for survival if in the ICU, 0.44 for survival on the ward).

**Table 2:** Physicians’ estimated probabilities of survival for 201 patients evaluated for intensive care

Estimate	Prediction by intensive care physician		Prediction by internist	
	Survival if care in the ICU *N* (%)	Survival if care on the ward *N* (%)	Survival if care in the ICU (2 missing) *N* (%)	Survival if care on the ward *N* (%)
<10%	15 (7.5)	72 (35.8)	8 (4.0)	62 (30.8)
10–40%	34 (16.9)	47 (23.4)	19 (9.5)	62 (30.8)
41–60%	33 (16.4)	32 (15.9)	37 (18.6)	49 (24.4)
61–90%	54 (26.9)	28 (13.9)	72 (36.2)	24 (11.9)
> 90%	65 (32.3)	22 (10.9)	63 (31.7)	4 (2.0)
Within physician*	*P* < 0.001		*P* < 0.001	

^*^Wilcoxon paired test

### Estimated survival benefit

We examined the difference in expected survival categories between care in the ICU and on the ward (Table 3). For the ICU physicians, 31.4% of the patients would not increase their chances of survival if admitted to the ICU. The other patients would gain between 1 and 4 categories on the 5-level survival scale. Internists were globally more optimistic than intensive care physicians (*P* < 0.001) and classified only 11.9% of the patients as not increasing their chances of survival if admitted to the ICU.

**Table 3:** Estimated gain in survival attributable to intensive care, and observed proportion of patients admitted

Difference in survival categories* between ICU and ward	Intensive care physician		Internist	
	*N* (%)	Proportion admitted (%)	*N* (%)	Proportion admitted (%)
Loss of 1	5 (2.5)	80.0	–	
Even	58 (28.9)	32.8	24 (11.9)	37.5
Gain of 1	62 (30.8)	79.0	75 (37.3)	61.3
Gain of 2	50 (24.9)	88.0	65 (32.3)	86.2
Gain of 3	22 (10.9)	90.9	29 (14.4)	82.8
Gain of 4	4 (2.0)	100	6 (3.0)	83.3
Test for linear trend		<0.001		<0.001

*Survival was categorized as < 10%, 10–40%, 41–60%, 61–90%, > 90%

### Survival benefit and admission to the ICU

Ratings by both physicians were associated with observed proportions of admitted patients (Table 3). The probability of admission was below 40% for patients who were not expected to improve their survival if admitted, but exceeded 80% if the patient was predicted to gain 2 categories of survival or more. The linear association between survival gain and admission was highly significant for both physicians. However, ICU physicians’ estimates had a greater influence on the admission decision: the adjusted odds ratios of admission were 7.6 (95% confidence interval 3.6–15.8) for a gain of 1–2 categories, and 30.8 (95% CI 3.8–247) for a gain of 3–4 categories, compared to no gain. The corresponding adjusted odds ratios for estimates made by the internist were 2.3 (95% CI 0.8–6.5) and 4.2 (95% CI 1.1–16.4).

### Patient survival

Of the 140 patients admitted to the ICU, 40 (28.6%) had died at 28 days of follow-up, and of the 61 patients who were not admitted to the ICU, 18 (29.5%) had died. Observed survival was associated with predicted survival, for both groups of physicians (Table 4). Patient survival in the ICU ranged from 33.3 to 91.4% across categories of survival predicted by ICU physicians, and from 0 to 95.1% across predictions made by internists. The discrimination of the predictions, measured by the area under the ROC curve, was 0.63 and 0.76, respectively. The predictions for the patients who stayed on the ward were of similar accuracy. The gradients of observed proportions of survivors ran from 40.0 to 85.0% across estimates made by ICU physicians, and from 25.0 to 100% across estimates made by internists. The areas under the ROC curves were 0.69 and 0.74, respectively.

**Table 4:** Patients’ observed 28 day survival according to physicians’ survival predictions

Predicted survival if care in the ICU	Patients admitted to intensive care (*N* = 140)			
	Intensive care physicians		Internists	
	*N* (column %)	Survived (row %)	*N* (column %)	Survived (row %)
<10%	6 (4.3)	2 (33.3)	2 (1.4)	0 (0)
10–40%	30 (21.4)	18 (60.0)	16 (11.4)	4 (25.0)
41–60%	27 (19.3)	18 (66.7)	28 (20.0)	17 (60.7)
61–90%	42 (30.0)	30 (71.4)	53 (37.9)	40 (75.5)
> 90%	35 (25.0)	32 (91.4)	41 (29.3)	39 (95.1)
*P* for linear trend	*P* = 0.001		*P* < 0.001	
Area under ROC curve	0.63 (0.53–0.73)		0.76 (0.67–0.84)	

Predicted survival if care on the ward	Patients NOT admitted to intensive care (*N* = 61)			
	Intensive care physicians		Internists	
	*N* (column %)	Survived (row %)	*N* (column %)	Survived (row %)
<10%	10 (16.4)	4 (40.0)	12 (19.7)	3 (25.0)
10–40%	11 (18.0)	7 (63.6)	14 (23.0)	11 (78.6)
41–60%	9 (14.8)	7 (77.8)	19 (31.1)	15 (78.9)
61–90%	11 (18.0)	8 (72.7)	13 (21.3)	11 (84.4)
> 90%	20 (32.8)	17 (85.0)	3 (4.9)	3 (100)
*P* for linear trend	*P* = 0.016		*P* = 0.001	
Area under ROC curve	0.69 (0.54–0.84)		0.74 (0.61–0.89)	

We compared the predictions made by the two groups of physicians in mutually adjusted logistic regression models (Table 5). For both groups of patients—those admitted to the ICU and those who stayed on the ward—the prediction of the internists was more accurate. In fact, once the opinion of the internists was taken into account, the ICU physicians’ estimates did not increase the accuracy in predicting survival.

**Table 5:** Multivariate regression model of survival at 28 days, according to physicians’ baseline predictions

Physicians’ predictions	Odds ratio^*^	95% CI	*P* value
	*Patients admitted to ICU*		
Intensive care physicians	1.32	0.90–1.93	0.15
Internists	3.05	1.87–4.95	<0.001
	*Patients on ward*		
Intensive care physicians	1.23	0.78–1.96	0.37
Internists	2.23	1.14–4.34	0.019

*The odds ratios are for a 1 category increase on the 5-point survival prediction scale. Predictions made by internists and by ICU physicians are adjusted for each other

On the whole, physicians felt rather confident about their survival estimates, ICU physicians more so than internists (in the ICU: mean 4.2 vs. 3.9, on the ward 4.2 vs. 3.8, both *P* < 0.001). Physicians’ confidence differed according to the survival probabilities (Additional file 2: Table S2). It was higher for extreme probabilities (i.e., < 10% or > 90%) than for mid-range probabilities.

## Discussion

In this study we found that physicians involved in decisions to admit patients to the ICU were able to predict short term survival for admitted and non-admitted patients. The physicians perceived a survival benefit from an admission to intensive care for a majority of patients. Internists’ estimates of survival were higher than ICU physicians’, but globally there was a substantial agreement between the 2 groups of physicians. Physicians showed high levels of confidence in their estimates.

The estimated survival benefit was associated with the decision to admit a patient to the ICU: most patients with a high expected benefit were admitted compared to a minority of patients with a low expected benefit. The ICU admission decision was more strongly influenced by the ICU physician’s survival prediction than by the internist’s. However internists predicted patient survival more accurately than ICU physicians, both for patients admitted to the ICU and for those who remained on the ward.

Our results expand on available data. In a small study based on scenarios, good agreement about ICU survival prediction was also found between internists and ICU physicians, but their prediction accuracy varied [9]. In our study higher accuracy in prediction is likely explained by the fact that physicians estimated survival for patients they had actually assessed. We could not compare physicians’ performance with a scoring system as no validated score exists for estimating patient prognosis at the time of triage [10, 11]. However, physicians’ clinical judgment has been shown to be at least as accurate as objective risk scores for predicting mortality in patients admitted to the ICU [5] and for predicting medical in-patients’ deterioration [12]. Values of the ROC curves in our study were similar to those published for ICU physicians’ survival estimates for patients admitted to intensive care. Our findings also show that both internists and ICU physicians are accurate about patient prognosis under different circumstances, i.e., at the time of triage and for continued care on the ward or for care in the ICU.

In keeping with recommendations about ICU admission [1], physicians’ predicted gain in survival was associated with admission decisions. Interestingly, some patients with no expected survival benefit were admitted. It suggests that physicians are aware of their limited ability in predicting survival for individuals and prefer to err toward overtriage, an attitude supported by current guidelines [1]. Considerations other than survival may also be deemed important enough for physicians to propose intensive care. Notably, during the updating process of a consensus statement about triage principles, ICU experts could not agree about a survival cutoff precluding ICU admission, not even for a chance of survival of 0.1% or less [13]. Furthermore, ICU physicians seem to use less stringent admission criteria when more ICU beds are available [14–16].

Not surprisingly the ICU physicians’ opinions weighed more than the internists’ in the decision to admit a patient to the ICU. ICU physicians are considered experts and most capable in estimating the benefit of intensive care for patients. Our findings however challenge this assumption, as internists were more accurate in predicting patient survival for both care on the ward and in the ICU. In this respect, the influence of ICU physicians on the admission decision may not be entirely justified, all the more so that they sometimes assume a dismissive behavior toward requesting physicians and potentially disregard their assessment [8, 17, 18]. Because considerations other than survival benefit influence ICU admission decisions, increased collaborative decision making between ICU physicians and internists seems advisable.

Our study has several limitations. The involved physicians were contacted after they had discussed intensive care for a patient. So their opinions were not independent as they had had the opportunity to come to a shared assessment of the situation. It may partially explain the high level of agreement between the physicians. In some cases, the patient may have died by the time the physicians completed the questionnaire, in which case survival predictions would have been meaningless. However, it would not change the main findings of this study, as it would concern a minority of patients. Moreover, only the physician caring for the patient would know about his death, and for patients assessed during night shifts and week-ends he would not necessarily be the physician in charge at the time of death. Delay between admission decision and completion of the questionnaire may cause an overestimation of physicians’ accuracy in prediction, as the physician in charge had the opportunity to observe how the patient’s condition evolved. We minimized this risk by contacting physicians twice a day, and by offering them to complete the questionnaire on the phone, thus keeping delays as short as possible. Moreover, on average, the delays were longer for ICU physicians, whose predictions were less accurate than those of the internists. Hence, if delays caused a bias, it should be conservative.

## Conclusions

Physicians are able to predict survival probabilities for patients assessed for admission to intensive care, albeit imperfectly. Internists are more accurate than ICU physicians. However, ICU physicians’ estimates more strongly influence the admission decision. These results highlight the need for objective risk scores in support to physicians’ judgment and for closer collaboration between internists and ICU physicians. Strategies to improve the decision making process should focus on the development of valid triage scoring systems and address potential communication gaps between physicians.

## Additional files

**Additional file 1: Table S1.** Characteristics of ICU physicians and internists.

**Additional file 2: Table S2.** Physicians’ mean confidence rating across predictions of survival on a 5-point scale.
