# Supplementary material for: ICU physicians’ and internists’ survival predictions for patients evaluated for admission to the intensive care unit
Source: Ann Intensive Care. 2018 Nov 14;8:108. doi: 10.1186/s13613-018-0456-9 (PMC6236006; doi:10.1186/s13613-018-0456-9)
Supplement: Supplementary file 1 — Additional file 1: Table S1. Characteristics of ICU physicians and internists. [file 13613_2018_456_MOESM1_ESM.docx]

Table S1. Characteristics of ICU physicians and internists

| Characteristics | ICU physicians  (n=30) | Internists  (n=97) | |  |
| --- | --- | --- | --- | --- |
| Sex (n)  Men  Women | 20  10 | 38  59 | |  |
| Age (mean; range) | 38 (25 – 59) | 30 (24 – 43) | |  |
| Professional position (n)  Attending physician  Chief resident  Resident | 6  24  0 | 1  21  75 | |  |
| Years since graduation (mean; range) | 12 (4 – 32) | 7 (0 – 28) | |  |
| Years in the current position (mean; range) | 4 (0.1 – 21) | 2 (0.1 – 6) | |  |
| Years of ICU / internal medicine experience (mean; range) | 7 (0.25 – 25) | 3.5 (0.1 – 18) | |  |
|  |  | |  | |
