# Supplementary material for: ICU physicians’ and internists’ survival predictions for patients evaluated for admission to the intensive care unit
Source: Ann Intensive Care. 2018 Nov 14;8:108. doi: 10.1186/s13613-018-0456-9 (PMC6236006; doi:10.1186/s13613-018-0456-9)
Supplement: Supplementary file 2 — Additional file 2: Table S2. Physicians’ mean confidence rating across predictions of survival on a 5-point scale. [file 13613_2018_456_MOESM2_ESM.docx]

Table S2. Physicians’ mean confidence rating across predictions of survival on a 5-point scale.

| Predicted survival | Intensive care physicians | | Internists | |
| --- | --- | --- | --- | --- |
|  | Survival in ICU | Survival on ward | Survival in ICU | Survival on ward |
| <10% | 4.9 | 4.7 | 4.5 | 4.5 |
| 10-40% | 3.8 | 3.8 | 3.5 | 3.6 |
| 41-60% | 3.8 | 3.7 | 3.4 | 3.4 |
| 61-90% | 3.8 | 4.1 | 3.8 | 3.7 |
| >90% | 4.7 | 4.8 | 4.3 | 4.0 |
